# What are weight bias measures measuring? An evaluation of core measures of weight bias and weight bias internalisation

**DOI:** 10.1177/20551029211029149

**Published:** 2021-07-28

**Authors:** Sarah-Jane F Stewart, Jane Ogden

**Affiliations:** University of Surrey, UK

**Keywords:** beliefs, health psychology, illness perception, obesity, stigma

## Abstract

Research exploring weight bias and weight bias internalisation (WBI) is grounded upon several core measures. This study aimed to evaluate whether operationalisations of these measures matched their conceptualisations in the literature. Using a ‘closed card-sorting’ methodology, participants sorted items from the most used measures into pre-defined categories, reflecting weight bias and non-weight bias. Findings indicated a high degree of congruence between WBI conceptualisations and operationalisations, however found less congruence between weight bias conceptualisations and operationalisations, with scale-items largely sorted into non-weight bias domains. Recommendations for scale modifications and developments are presented alongside a new amalgamated weight bias scale (AWBS).

## Introduction

Despite the consistency of research documenting the negative relationships between weight bias, weight bias internalisation (WBI) and various health-related outcomes (Jackson et al., 2015; Pearl and Puhl, 2018; Puhl and Brownell, 2001), whether measures of these constructs actually capture what they are aiming to measure remains a contentious issue and underpins research in this area (Meadows and Higgs, 2019).

Research has outlined the need for conceptualisations of weight bias and WBI to be articulated clearly (DePierre and Puhl, 2012; Lacroix et al., 2017; Stewart and Ogden, 2019). Weight bias is poorly conceptualised within the literature, and although research has validated the most commonly used WBI measurement scales, their psychometric properties are inconsistent (Pearl and Puhl, 2018). Therefore, it is necessary for research to establish whether scales for weight bias and WBI truly represent those constructs and are appropriate measurement tools.

### Defining weight bias

Despite consensus that weight bias is affectively negative, there remains some variation in how it is defined. Tomiyama (2014) defines weight stigma to be ‘the social devaluation and denigration of people perceived to carry excess weight and leads to prejudice, negative stereotyping and discrimination towards those people’. However, Lacroix et al. (2017) used the framework outlined by Cook et al. (2014) to outline three ‘categories’ of weight bias, including structural, interpersonal and intrapersonal (internalised) weight bias.

The blame and controllability of obesity are considered to be key components of weight bias, and whilst relationships are implied, they are not typically outlined in definitions. For example, Puhl et al. (2015) describe personal blame and responsibility for body weight and related stereotypes as contributing factors to weight bias, rather than weight bias itself.

Despite subtle variances across existing definitions, there is consensus that weight bias can broadly be defined as negative attitudes, manifested in negative stereotypes towards those perceived to be affected by overweight or obesity (e.g. beliefs that persons with overweight and obesity are lazy, sloppy, incompetent and lack willpower; (Pearl and Puhl, 2018; Puhl and Brownell, 2001; World Health Organization [WHO], 2017). Due to the notable recognition this definition has received both in terms of research (Pearl and Puhl, 2018) and practical applications (WHO, 2017), it is the definition underpinning this research.

### Defining WBI

Although there are still some variations in how WBI is conceptualised, it is comparably more straightforward than weight bias. Durso and Latner (2008), and the WHO (2017) define WBI as ‘holding negative beliefs about oneself due to weight or size’. Comparably, Pearl and Puhl (2018) define WBI as, ‘the internalisation of negative weight stereotypes and subsequent self-disparagement’. Corrigan et al. (2006) marry these key intrapersonal features in a more comprehensive definition; (i) awareness of negative stereotypes about one’s social identity; (ii) agreement with and application of those stereotypes to oneself; and (iii) self-devaluation as a result. This definition is therefore used as the conceptual underpinnings of WBI in this research, reflecting broad consensus across the literature (Pearl and Puhl, 2018).

### The present study

Research has reviewed the measures of weight bias and WBI, highlighting them to be accessible, hold ‘adequate’ internal consistency, and draw upon the key dimensions of each construct (Lacroix et al. 2017; Ruggs et al., 2010). However, research has yet to empirically examine whether these operationalisations map onto conceptualisations within the literature. This study aimed to build on the previous works by Lacroix et al. (2017) and Ruggs et al. (2010) and investigate whether operationalisations of weight bias and WBI match conceptualisations of these constructs using leading measures of weight bias and WBI in two studies. As these scales are designed for use within the general population; a general population sample was used to carry out the analysis.

The literature often uses terms such as weight bias and weight/obesity stigma interchangeably. In this study, the term weight bias is used throughout.

## Study 1: Weight bias

### Methods

#### Design

This study design resembled an online ‘closed card-sorting task’ (Fincher and Tenenberg, 2005; Rugg and McGeorge, 2005). Participants sorted scale-items (‘cards’) from the five most used weight bias scales into a set of pre-defined categories reflecting weight bias and non-weight bias domains.

#### Participants

A total of 189 participants from the general population were recruited via online opportunity sampling. The mean age of participants was 30.0 (SD = 12.6, range 17–73); 77.3% were female (*n* = 146), 21.2% were male (*n* = 40) and 1.5% classified as other (*n* = 3). Most participants were white (79.4%, *n* = 150), 9.5% were Asian (*n* = 18), 4.8% were Black (*n* = 9) and 6.4% classified as other (*n* = 12). Participants provided self-reported BMI classifications; 3.7% were underweight (*n* = 7), 72.5% were healthy weight (*n* = 137), 16.4% reported being overweight (*n* = 31) and 7.4% reported having obesity (*n* = 14).

A minimum of 15 participants is generally considered appropriate for studies using a card-sort methodology (Nielsen, 2004). However, given individual differences and the widespread nature of weight bias (Pearl and Puhl, 2018; Puhl and Brownell, 2001), it was important to aim to recruit a heterogenous population to have a sense of generalisability and representativeness. Therefore, based on early card-sort research, a sample of 150–200 participants was deemed appropriate given our study aims (McCauley et al., 2005; Sachs and Josman, 2003).

#### Materials

##### Measures of weight bias

Numerous measures of weight bias are in circulation that vary in their psychometric properties (Lacroix et al., 2017). However, our goal is not to produce a fully comprehensive evaluation of the degree to which conceptualisations map onto operationalisations for *all* measures of weight bias; but rather of those self-report measures of weight bias considered to dominate the field.

The five most-cited weight bias scales were selected, established through their *Google Scholar* total citation-count and their inclusion within a systematic review of the psychometric characteristics and properties of weight bias scales (Lacroix et al., 2017). PsycINFO and Google Scholar databases were also searched for scales that either been missed or published since. Citation-by-year data was extracted from *Publish or Perish* to indicate whether any recently published scales were receiving particularly high numbers of citations, however this data suggested that they were not. The scales and their citation count as of May 2021 were as follows:

*Anti-fat attitudes questionnaire* (AFA; Crandall, 1994). This 13-item questionnaire assesses explicit stigma and comprises of three subscales: dislike (explicit antipathy toward persons with obesity); fear of fat (personal concern overweight); and willpower (the extent to which obesity is believed to be attributable to an individual’s personal control). Citation count = 1886.
*Attitudes towards obese people* (ATOP; Allison et al., 1991). This 20-item questionnaire measures stereotypical attitudes about persons with obesity, inclusive of perceptions about their self-esteem, personality and social quality of life, and was based on the attitudes towards disabled persons scale (ATDP; Yuker and Block, 1986). Citation count = 441.
*Beliefs about obese people* (BAOP; Allison et al., 1991). This 8-item questionnaire measures beliefs surrounding the causes and controllability of obesity. Citation count = 441.
*Obese persons trait survey* (OPTS; Puhl et al., 2005). This 20-item scale includes 10 negative traits and 10 positive traits and asks participants to estimate the percentage of persons with obesity that possess them. Citation count = 357.
*Anti-fat attitudes scale* (AFAS; Crandall and Biernat, 1990). This 5-item scale measures attitudes surrounding controllability and fear of fat. Citation count = 363.

##### Demographics

Participants reported information relating to their age, gender, ethnicity and their self-reported BMI group.

##### The categories for matching

Seven categories were formulated on the basis of current conceptualisations of weight bias (Pearl and Puhl, 2018; WHO, 2017), measure subscales (Allison et al., 1991; Crandall, 1994; Crandall and Biernat, 1990; Puhl et al., 2005), and wider evidenced domains relating to obesity (Puhl et al., 2015). The seven weight bias and non-weight bias categories and their definitions were; Weight bias categories: (i) ‘Dislike people with obesity = negative feelings towards those who are overweight’; (ii) ‘Fear of fat = negative feelings towards any fat on your own body’; (iii) ‘Negative stereotypes about people with obesity= negative characteristics that a lot of people feel represent those who are overweight’; (iv) ‘Positive stereotypes about obese people= positive characteristics that a lot of people feel represent those who are overweight’: Non weight bias categories: (v) ‘Perceived causes of obesity’; (vi) ‘Perceived consequences of obesity’; (vii) ‘Perceived solutions to obesity’; Participants were also given the option ‘Other’.

#### Procedure

Using an online survey, participants provided their informed consent, and basic demographic information (age, gender, ethnicity and self-reported BMI group). Participants were then asked to sort each scale-item for each of the five weight bias scales into one of the seven categories they felt best described it.

Both studies included in this paper are compliant with institutional ethical guidelines set by the University of Surrey Ethics Committee (Ref no. 353003-352994-40934146).

### Results

Frequency counts and percentages for each item within each scale were calculated to provide the distribution of categories that items were sorted into. Table 1 provides total frequency counts and percentages were calculated for each scale, and overall total frequencies and adjusted percentages.

**Table 1.** Total frequency counts and percentages of weight bias measures sorted into each category.

Scale	Non-weight bias			Weight bias				
	Causes	Consequences	Solutions	Fear of fat	Dislike	Negative stereotypes	Positive stereotypes	Other
AFA								
*N*	104	102	164	547	839	596	27	78
%	4.23	4.15	6.67	22.26	34.15	24.26	1.10	3.17
ATOP								
*N*	113	442	122	252	512	1208	870	257
%	2.99	11.69	3.23	6.67	13.54	31.96	23.02	6.80
BAOP								
*N*	876	123	40	29	55	296	67	26
%	57.94	8.13	2.65	1.92	3.64	19.58	4.43	1.72
OPTS								
*N*	335	331	197	74	207	1094	1349	190
%	8.86	8.76	5.21	1.96	5.48	28.94	35.69	5.03
AFAS								
*N*	63	118	53	283	126	259	21	21
%	6.67	12.49	5.16	29.95	13.33	27.41	2.22	2.22
Total *N*	1491	1116	576	1185	1739	3453	2334	572
Adjusted %	16.15	9.05	4.68	12.56	14.04	26.44	13.30	3.79

There were eight cases of missing data, which have been adjusted for in the final Total *N* and %.

**Figure 1.**
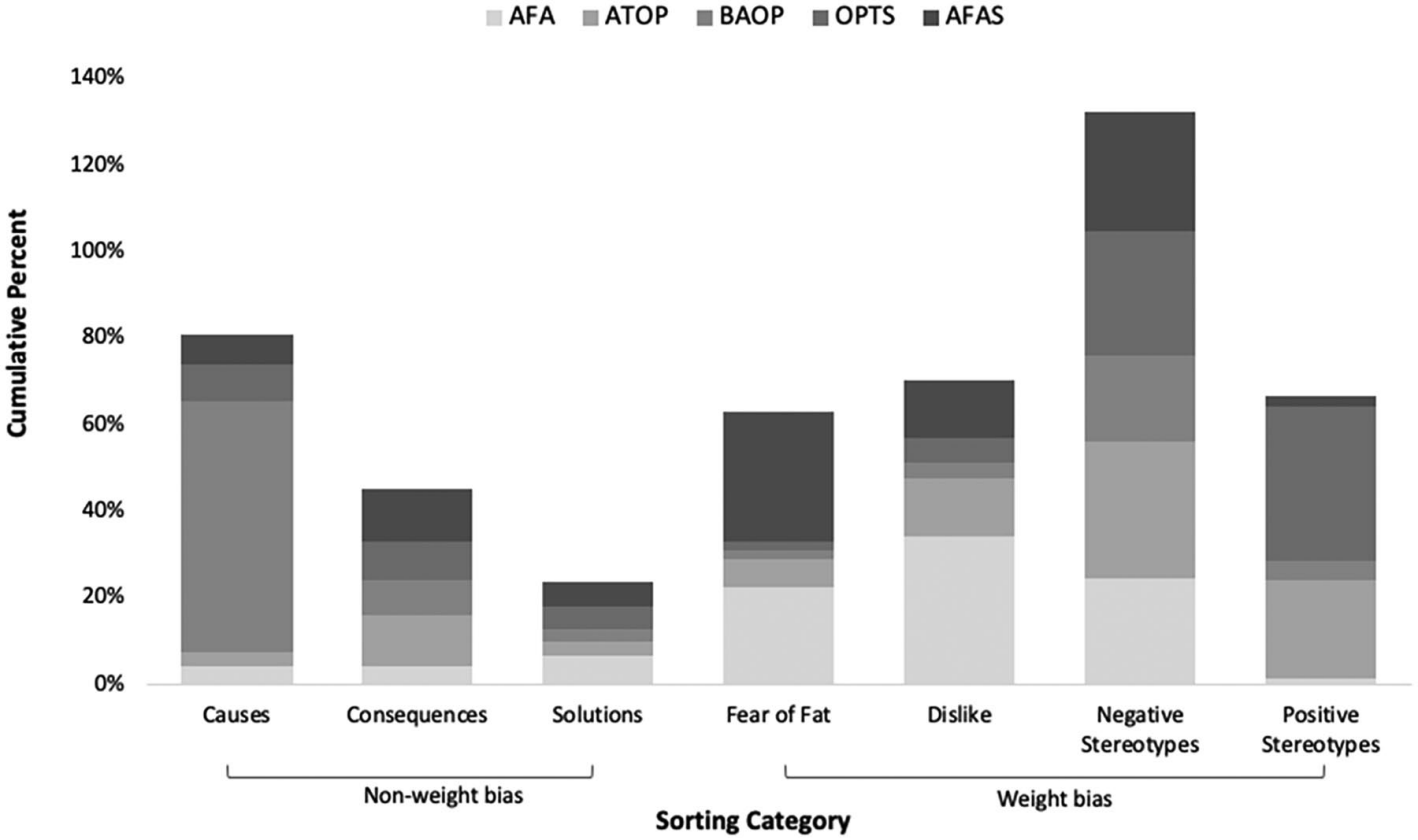
Cumulative percentages of weight bias scale-items sorted into each category.

Findings illustrate a wide variation in how each item from each scale was sorted into the categories. Figure 1 provides an overview of the cumulative percentages of scale-items sorted into each category.

Overall, the category that had the highest percentage of scale-items sorted into was ‘negative stereotypes’, and ‘Causes of obesity’ had the second highest percentage. The combined total number of items that were coded into categories across all scales was *N* = 12,466. The total number of items sorted into causes, consequences and solutions to obesity was *N* = 3183 (29.88%).

The total number of items coded under ‘Cumulative percentages of WBI scale-items sorted other’ was *N* = 572. From this, the total number of times participants provided an accompanying free-text response was *N* = 325. The research team systematically assessed each of these to establish whether it could be appropriately re-coded into any of the pre-defined categories. For example, a text response of ‘discrimination/prejudice’ would be re-coded into ‘negative stereotypes’ in accordance with the category definitions, this was done for a total of *N* = 48 (0.39%) responses.

#### AFA

The most common category that items from AFA were sorted into was ‘dislike people with obesity’. The combined total frequency for items sorted into causes, consequences and solutions to obesity was *N* = 370 (15.05%).

#### ATOP

The most common category that items from ATOP were sorted into was ‘negative stereotypes’. The combined total frequency for items sorted into causes, consequences and solutions to obesity was *N* = 677 (17.91%).

#### BAOP

The most common category that items from BAOP were sorted into was ‘causes of obesity’. The combined total frequency for items sorted into causes, consequences and solutions to obesity was *N* = 1039 (68.72%).

#### OPTS

The most common category that items from OPTS were sorted into was ‘positive stereotypes’. The combined total frequency for items sorted into causes, consequences and solutions to obesity was *N* = 863 (22.83%).

#### AFAS

The most common category that items from AFAS were sorted into was ‘fear of fat’. The combined total frequency for items sorted into causes, consequences and solutions to obesity was *N* = 243 (24.77%).

### Discussion

This study evaluated whether operationalisations of the most commonly used measures of weight bias matched conceptualisations within the literature. Whilst most scale-items were sorted to reflect conceptualisations of weight bias, a large percentage were sorted into categories reflecting non-weight bias domains. In particular, whilst ‘Negative stereotypes’ was the most commonly sorted category, in accordance with widely accepted conceptualisations of weight bias (Pearl and Puhl, 2018; Puhl and Brownell, 2001), the second most commonly sorted category was ‘causes of obesity’, a domain not typically in line with definitions of weight bias (Alberga et al., 2016; Pearl and Puhl, 2018; Tomiyama, 2014; Washington, 2011). This was followed by ‘dislike’, ‘positive stereotypes’ and ‘fear of fat’. The least commonly sorted categories were ‘consequences’ and ‘solutions’; domains also not in accordance with definitions of weight bias. It is therefore concluded that current operationalisations of weight bias do not entirely match the conceptualisation of weight bias, indicating that existing measures of weight bias measure both weight bias and non-weight bias domains.

## Study 2: WBI

### Methods

#### Design

The research design for this study was the same as that outlined in Study 1.

#### Participants

A total of 168 participants completed the questionnaire. The mean age of participants was 29.8 (SD = 11.5, range 18–71); 69.6% were female (*n* = 117), and 30.4% were male (*n* = 51). Most participants were white (81.5%, *n* = 137), 10.1% were Asian (*n* = 17), 3.6% were Black (*n* = 6) and 4.8% classified as other (*n* = 8). Participants reported self-reported BMI classifications; 5.4% were underweight (*n* = 9), 68.5% were healthy weight (*n* = 115), 21.4% reported being overweight (*n* = 36) and 4.8% reported being obese (n=8).

#### Materials

##### Measures of WBI

According to citation count and a systematic review investigating the relationship between WBI and health (Pearl and Puhl, 2018), the literature is heavily dominated by two scales assessing WBI which were therefore included in this study:

*Weight Bias Internalisation Scale* (WBIS; Durso and Latner, 2008). This 11-item questionnaire assesses the degree of various domains of internalised weight bias within persons with overweight and obesity. Citation count = 461.
*Weight Self-Stigma Questionnaire* (WSSQ; Lillis et al., 2010). This 12-item questionnaire assesses weight self-stigma and was designed to capture the multi-dimensional nature of WBI. The WSSQ comprises of two distinct subscales: self-devaluation, and fear of enacted stigma. Citation count = 190.

##### Demographics

Participants reported their age, gender, ethnicity and their self-reported BMI group.

##### The categories for matching

WBI is conceptualised as (i) awareness of negative stereotypes about one’s social identity; (ii) agreement with and application of those stereotypes to oneself; and (iii) self-devaluation as a result (Corrigan et al., 2006; Pearl and Puhl, 2018). The categories were derived from the domains and subscales that WBI scales draw upon (Durso and Latner, 2008; Lillis et al., 2010) and wider evidence documenting the relationship between weight and behaviour (Pearl and Lebowitz, 2014). This led to the creation of three categories reflecting both weight bias and non-weight bias domains: Weight bias: (i) high (or low) fear of criticism from others due to weight; (ii) high (or low) self-criticism due to weight; Non-weight bias: (iii) weight is related to behaviour.

#### Procedure

The procedure for Study 2 was the same as for Study 1 but with the use of items from measures of WBI to be sorted into the new set of three categories. The questionnaire took between 5 and 10 minutes to complete.

### Results

Total frequency counts and percentages of the items sorted into each of the categories were calculated for each scale. Table 2 presents overall total frequencies and adjusted percentages.

**Table 2.** Total frequency counts and percentages of WBI measures sorted into each category.

Scale	Non-weight bias	Weight bias	
	Behaviour	Fear from others	Self-criticism
WBIS			
*N*	177	180	775
%	15.64	15.90	68.46
WSSQ			
*N*	206	487	578
%	16.21	38.32	45.48
Total *N*	383	667	1353
Adjusted %	15.92	27.11	56.97

**Figure 2.**
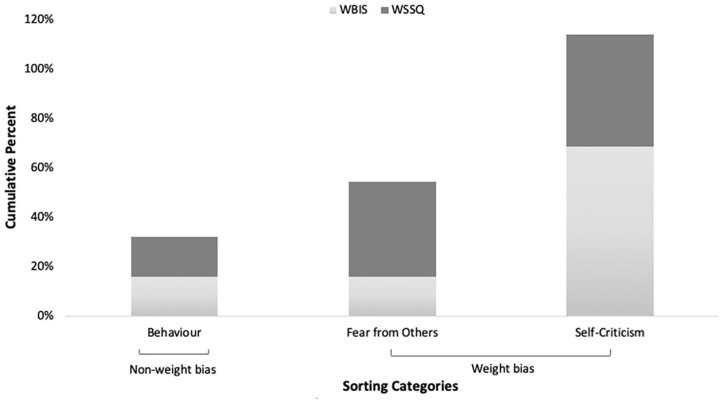
Cumulative percentages of WBI scale-items sorted into each category.

Figure 2 provides an overview of the cumulative percentages of scale-items sorted into each category.

The category that had the highest cumulative percentage of scale-items sorted into was ‘high/low self-criticism due to weight’.

#### WBIS

Completed data was received from 103 participants. Findings demonstrated that the most common category for participants to sort items from WBIS into was ‘high/low self-criticism due to weight’.

#### WSSQ

Completed data was received from 106 participants. The most common category for participants to sort items from WSSQ into was ‘high/low self-criticism due to weight’.

### Discussion

This second study explored whether operationalisations of the most commonly used measures of WBI match conceptualisations within the literature. Findings demonstrate that measures of WBI are clearly matched with the conceptualisations of WBI. In particular, the most common category for scale-items to be sorted into was ‘high/low self-criticism due to weight’, tapping into the key dimensions of definitions of WBI such as awareness of, and agreement with negative stereotypes and self-devaluation as a result (Corrigan et al., 2006). This indicates that these two most common measures of WBI are measuring what they aim to measure.

## General discussion

Findings suggest that whilst weight bias is currently conceptualised in terms of negative attitudes and stereotypes (Pearl and Puhl, 2018; Puhl and Brownell, 2001; WHO, 2017), this is not reflected in its operationalisations. Whilst some scale-items were deemed to reflect stereotypes, many others we considered to reflect other non-weight bias domains including causes, consequences and solutions. In contrast, the results from the analysis of WBI were more encouraging, with most scale-items in line with WBI conceptualisations.

There are some problems with this study, however, that need to be addressed. It should be noted that our sample was recruited online and lacked racial and ethnic diversity. This limits the generalisability of the findings to a broader population. Further, many of the measures, particularly those assessing weight bias, do not adopt person-first language. Since the development of these scales, advances in research investigating the impact of weight bias have emphasised the importance of using person-first language, to ensure that those with obesity do not feel dehumanised (Kyle and Puhl, 2014; Meadows and Daníelsdóttir, 2016). Considering these scales are designed for use within populations with obesity, it is important that efforts are made to minimise further discrimination.

Consequently, it is suggested that future research should ensure that both conceptualisations and operationalisations of weight bias and WBI are clarified and aligned to improve the validity of research in this field. Interestingly, several of the weight bias scales highlighted in this study to be potentially problematic including the AFA (Crandall, 1994), ATOP (Allison et al., 1991) and OPTS (Puhl et al., 2005) are considered to be among the most psychometrically strong (Lacroix et al., 2017). Therefore these findings should be used in conjunction with Lacroix et al. (2017), Pearl and Puhl (2018) and Ruggs et al. (2010) when selecting measures of weight bias and WBI. Despite an already comprehensive database of measurement scales, these existing scales could be modified to ensure that operationalisations are consistent with conceptualisations. Alternatively, the development of new, carefully crafted scales could help to ensure these constructs are measured more accurately. This research therefore concludes with recommendations for the modification of existing scales to increase the congruence between operationalisations and conceptualisations of weight bias and WBI. Tables 3 and 4 present a summary of the items included within each of the weight bias (Table 3) and WBI (Table 4) scales, and the domains they relate to according to our findings. These items have been re-phrased where appropriate, to reflect person-first language (Kyle and Puhl, 2014; Meadows and Daníelsdóttir, 2016). It is hoped that these tables provide a useful toolkit for researchers to select measurement scales that accurately reflect the conceptualisation of these constructs.

**Table 3.** Recommendations for item selection for measurement scales of weight bias depending on domain being measured.

Scale	Weight bias				Non-weight bias			Items with no overall consensus
	Dislike persons with obesity	Negative stereotypes	Positive stereotypes	Fear of fat	Causes	Consequences	Solutions	
AFA	I really don’t like people with obesity much. I don’t have many friends that have obesity. I have a hard time taking people with obesity too seriously. People with obesity make me somewhat uncomfortable. If I were an employer looking to hire, I might avoid hiring a person with obesity.	I have a hard time taking people with obesity too seriously. Some people have obesity because they have no willpower. People with obesity tend to be obese pretty much through their own fault.		I feel disgusted with myself when I gain weight. One of the worst things that could happen to me would be if I gained 25 pounds. I worry about becoming obese.			People who weigh too much could lose at least some part of their weight through a little exercise.	I tend to think that people who are overweight are a little untrustworthy.
ATOP	Most people without obesity would not want to marry anyone who has obesity. Most people feel uncomfortable when they associate with people with obesity. People with obesity should not expect to lead normal lives.	Most people with obesity feel that they are not as good as other people. Most people with obesity are more self-conscious than other people. Workers with obesity cannot be as successful as other workers. People with severe obesity are usually untidy. Most people with obesity have different personalities than people without obesity. Most people with obesity resent people without obesity. People with obesity are more emotional than people without obesity. People with obesity tend to have family problems.	People with obesity are as happy as people without obesity. People with obesity are usually sociable. Most people with obesity are not dissatisfied with themselves. People with obesity are just as self-confident as other people. People with obesity are often less aggressive than people without obesity. People with obesity are just as healthy as people without obesity. People with obesity are just as sexually attractive as people without obesity.	One of the worst things that could happen to a person would be for him to develop obesity.				Most people with obesity are more self-conscious than other people.
BAOP					Obesity often occurs when eating is used as a form of compensation for lack of love or attention. In many cases, obesity is the result of a biological disorder. Obesity is caused by overeating. Most people with obesity cause their problem by not getting enough exercise. Most people with obesity eat more than people without obesity. The majority of people with obesity have poor eating habits that lead to their obesity. Obesity is rarely caused by a lack of willpower. People can be addicted to food, just as others are addicted to drugs, and these people usually develop obesity.			
OPTS		Lazy Undisciplined Gluttonous Self-Indulgent Un-clean Lack of Willpower Unattractive Insecure	Honest Generous Sociable Productive Organised Friendly Outgoing Intelligent Warm Humorous					Unhealthy Sluggish
AFAS		People who have little control over their weight probably have little control over the rest of their lives. Nobody needs to have obesity. If they are, it’s probably because they eat too much or don’t exercise enough.		One of the worst things that could happen to me would be if I gained 25 pounds. Having obesity is one of the worst things a person can do to his or her health.				(I would like my child to be. . .) of normal weight.

**Table 4.** Recommendations for item selection for measurement scales of WBI depending on domain being measured.

Scale	WBI		Items with no overall consensus
	High/low fear of criticism from others	High/low self-criticism due to weight	
WBIS	I feel anxious about being overweight because of what people might think of me.	I am less attractive than most other people because of my weight. I wish I could drastically change my weight. Whenever I think a lot about being overweight, I feel depressed. I hate myself for being overweight. My weight is a major way that I judge my value as a person. I don’t feel that I deserve to have a really fulfilling social life, as long as I’m overweight. I am OK being the weight that I am. Because I’m overweight, I don’t feel like my true self. Because of my weight, I don’t understand how anyone attractive would want to date me.	As a person who is overweight, I feel that I am just as competent as anyone.
WSSQ	I feel insecure about others’ opinions of me. People discriminate against me because I’ve had weight problems. It’s difficult for people who haven’t had weight problems to relate to me. Others will think I lack self-control because of my weight problems. People think that I am to blame for my weight problems. Others are ashamed to be around me because of my weight.	I’ll always go back to being a person that is overweight. I caused my weight problems. I feel guilty because of my weight problems. I became overweight because I’m a weak person. I would never have any problems with weight if I were stronger. I don’t have enough self-control to maintain a healthy weight.	

In addition, based on the present analysis, this paper presents a new amalgamated weight bias scale (AWBS). The new amalgamated weight bias scale (AWBS) and scoring instructions are presented in the Supplemental materials and have been used in subsequent research (Stewart and Ogden, 2021a, 2021b).

## Conclusion

This research evaluated the degree to which measures of weight bias and WBI match the conceptualisation of these constructs. Whilst measures of WBI reflect current conceptualisations, this was not the case for measures of weight bias which also include non-weight bias components. Further work is therefore needed if weight bias is to continue to be a core part of research in this area.

## Supplemental Material

sj-pdf-1-hpo-10.1177_20551029211029149 – Supplemental material for What are weight bias measures measuring? An evaluation of core measures of weight bias and weight bias internalisation Click here for additional data file. Supplemental material, sj-pdf-1-hpo-10.1177_20551029211029149 for What are weight bias measures measuring? An evaluation of core measures of weight bias and weight bias internalisation by Sarah-Jane F Stewart and Jane Ogden in Health Psychology Open
