# Supplementary material for: What are weight bias measures measuring? An evaluation of core measures of weight bias and weight bias internalisation
Source: Health Psychol Open. 2021 Jul 28;8(2):20551029211029149. doi: 10.1177/20551029211029149 (PMC8323429; doi:10.1177/20551029211029149)
Supplement: sj-pdf-1-hpo-10.1177_20551029211029149 – Supplemental material for What are weight bias measures measuring? An evaluation of core measures of weight bias and weight bias internalisation [file sj-pdf-1-hpo-10.1177_20551029211029149.pdf]

Supplementary materials

The Amalgamated Weight Bias Scale (AWBS).

		Strongly disagree				Strongly agree
1.	I really don't like people with obesity much.	1	2	3	4	5
2.	People with obesity tend to be obese pretty much through their own fault.	1	2	3	4	5
3.	I don't have many friends that have obesity.	1	2	3	4	5
4.	People with obesity make me somewhat uncomfortable.	1	2	3	4	5
5.	People with obesity lack willpower	1	2	3	4	5
6.	Most people without obesity would not want to marry anyone who has obesity.	1	2	3	4	5
7.	People with obesity are undisciplined	1	2	3	4	5
8.	Most people feel uncomfortable when they associate with people with obesity.	1	2	3	4	5
9.	People with obesity should not expect to lead normal lives.	1	2	3	4	5
10.	Some people have obesity because they have no willpower.	1	2	3	4	5
11.	People with obesity are un-clean	1	2	3	4	5
12.	Workers with obesity cannot be as successful as other workers.	1	2	3	4	5
13.	People who have severe obesity are usually untidy.	1	2	3	4	5
14.	I have a hard time taking people with obesity too seriously.	1	2	3	4	5
15.	People with obesity are unattractive	1	2	3	4	5
16.	Most people with obesity resent healthy weight people.	1	2	3	4	5
17.	People with obesity are lazy	1	2	3	4	5
18.	People with obesity are gluttonous	1	2	3	4	5
19.	People who have little control over their weight probably have little control over the rest of their lives.	1	2	3	4	5
20.	If I were an employer looking to hire, I might avoid hiring a person with obesity.	1	2	3	4	5
21.	People with obesity are self-indulgent	1	2	3	4	5
22.	Nobody needs to have obesity. If they are, it's probably because they eat too much or don't exercise enough.	1	2	3	4	5
23.	People with obesity are insecure	1	2	3	4	5

Note. The Amalgamated Weight Bias Scale (AWBS) is a 23-item scale. It has been tested on a larger sample (N=1,201) as part of two subsequent studies (Stewart and Ogden, 2020; 2021), and resulted in two subscales; i) negative stereotypes ($\alpha = 0.92$) and dislike of people with obesity ($\alpha = 0.80$). To calculate the score for negative stereotypes, responses for items 2, 5, 7, 10, 11, 12, 13, 15, 16, 17, 18, 19, 21, 22, 23 should be summed. To calculate the score for dislike of people with obesity, responses for items 1, 3, 4, 6, 8, 9, 14, 20 should be summed. To calculate a total score for the AWBS, all scale-items should be summed. A higher score indicates higher weight bias.
